# Mechanical Thrombectomy by a Direct Aspiration First Pass Technique (ADAPT) in Ischemic Stroke: Results of Monocentric Study Based on Multimodal CT Patient Selection

**DOI:** 10.1155/2018/6192483

**Published:** 2018-11-01

**Authors:** Giuseppe Guzzardi, Bruno Del Sette, Carmelo Stanca, Andrea Galbiati, Massimiliano Cernigliaro, Alessandro Carriero, Alessandro Stecco

**Affiliations:** SC Radiologia Diagnostica ed Interventistica Azienda Ospedaliero-Universitaria “Maggiore della Carità”, Italy

## Abstract

**Introduction:**

Mechanical thrombectomy with ADAP-technique of ischemic stroke has been reported as fast and effective. Aim of this study is to evaluate imaging criteria as possible predictors of stroke severity, therapeutic success, and outcome.

**Materials and Methods:**

Patients (30) presenting from October 2015 to April 2017 with Emergent Large Vessel Occlusion of the anterior circulation were treated with ADAP-technique. 22 received also IV tPA; 8 underwent endovascular treatment only. Every patient was evaluated with noncontrast CT, multiphase angiography-CT, and perfusion CT. Clinical and radiological characteristics were measured. Good clinical outcome was an improvement of 8 points on NIHSS at discharge or a modified Rankin Scale ≤2 at discharge and at 90 days.

**Results:**

Successful revascularization was obtained in 57% of patients, no procedural complications were witnessed, and only two hemorrhages were reported. Good outcome at discharge was obtained in 11 patients (37%) and predicted by NCCT ASPECT and TICI; outcome at 90 days was predicted by NCCT ASPECT, clot length, and premorbid mRS. Mortality was 23% at discharge and 30% at 90 days.

**Conclusion:**

ADAPT is an effective endovascular method of stroke treatment with fast procedural times. Multimodal CT evaluation is effective in assessing stroke severity, providing important prognostic information, which is able to select patients for the appropriate treatment.

## 1. Introduction

Despite modern medicine advancement, on prevention and treatment of ischemic stroke, its incidence and its social impact are still severe. Although global stroke incidence is declining, rates observed in young adults are on the rise, thus suggesting a need for strategies to improve prevention. Overall, mortality in the first 4 weeks following an ischemic stroke is 20%, increasing to 30% in the first 12 months. Only 25% of patients who survive an ischemic stroke recover fully, while the majority become disabled, and in a substantial proportion of these, disability is so severe that they are no longer self-sufficient and need to be managed in chronic care settings [1, 2].

Therefore, new diagnostic and therapeutic strategies must be developed to improve global outcome of stroke patients.

After a cerebral ischemic event, outcomes could range from a complete functional recovery to death, with different degrees of disability in between; this variety of outcomes is mainly related to volume and location of the ischemic tissue, but also to the amount of time needed to reperfuse the tissue [3].

Main goal in treating ischemic stroke patients is to rescue the ischemic penumbra. The portion of hypothetical salvageable tissue is time dependent, but each patient presents a different amount of collateral blood flow, making the time window widely variable. Recent new trials have demonstrated that in selected patients salvageable tissue may survive up to 24h [4, 5].

Multimodal CT Stroke Protocol, including Noncontrast Computed Tomography (NCCT), CT-Angiography (CTA), and CT-Perfusion (CTP), fulfills all requirements for hyperacute stroke imaging [6], being able to acquire all needed information in a short time with conventional CT equipment, which is widely available and requires short scan times. This protocol allows, in short times, excluding other causes of symptoms, detecting exact vessel occlusion, estimating extent of collateral vessels, [7] and distinguishing infarct core from penumbra area [8–10]. After publication of recent randomized trials different approaches for treatment and diagnosis of stroke patients have been proposed. In our center, all patients were studied with a standardized multimodal CT protocol. Despite MRI superiority in assessing small and early ischemic changes in brain tissue, we opted for CT protocol due to machine availability; indeed in our institution MRI is in a building other than that of the emergency room; therefore patient transfer for imaging would take longer times.

Procedures were performed using “A Direct Aspiration first Pass Technique” (ADAPT); this therapeutic choice was made since it allowed only a proximal engagement of the clot, reducing procedural times without the need of distal microcatheterization of the occluded vessel.

We retrospectively evaluated all patients that underwent endovascular treatment for ischemic stroke from 2015 to assess efficacy and safety of ADAP-technique in stroke patients with emergent large vessel occlusion (ELVO) and evaluate whether clinical scores and scores found with multimodal brain imaging protocol could estimate stroke severity and outcome of treated patients.

## 2. Materials and Methods

We retrospectively evaluated every patient who underwent mechanical thrombectomy for ischemic stroke in the anterior circulation admitted in our Department of Interventional Radiology, from October 2015 to April 2017.

For each patient, clinical and radiological evaluation have been performed and data were collected in the main database.

Ethical review and approval were not required for this study in accordance with the national guidelines and institutional requirements.

### 2.1. Treatment Protocol

Patients arriving with acute stroke symptoms (AIS) at our facility (Hub) or a neighboring hospital (Spoke) were immediately evaluated by an expert neurologist, who collected National Institutes of Health Stroke Scale (NIHSS) score for each patient. Patients then underwent a CT scan and, after a hemorrhagic stroke was excluded, a Computed Tomography Angiography (CTA) and Computed Tomography Perfusion (CTP) scan were performed.

Patients without contraindications to intravenous treatment with tissue-type plasminogen activator (IV tPA) and within the right time window were treated accordingly.

Endovascular mechanical thrombectomy was then performed in patients with documented emergent large vessel occlusion (ELVO) at CTA.

### 2.2. Selection Criteria

All patients presenting with an ischemic stroke meeting the inclusion criteria for endovascular therapy were included in the study. Selection criteria for mechanical thrombectomy complied with an internal protocol (Table 1). During the time of study, we treated a total of 30 patients that were retrospectively evaluated.

### 2.3. Radiological Evaluation

A multidetector helical CT was used to scan head and neck (Brilliance 16-Slice, Philips Healthcare, Cleveland, OH): after, noncontrast CT of the brain (contiguous, 2-mm-thick sections, 120kV, 300mAs) CTA from aortic arch to vertex was obtained, with infusion of 90 mL of contrast material with 18–20 gauge IV at 4 mL/s plus 30mL of physiological saline at 4mL/s (20-second delay before imaging, section thickness 1 mm, 120 kV, 305 mAs). After the first angiographic acquisition, we then acquired two scans (protocol set as in basal CT) from C2 vertebra to vertex to assess the presence of delayed collateral vessels in the ischemic area.

Clot Burden Score (CBS) and Pial Arterial Filling Score (PAF) were calculated on CTA to estimate length of occlusion and collateral vessel recruitment.

CTP protocol included an initial unenhanced CT of the whole brain. Collimation was set at 6 mm. A section of 24 mm was then obtained, with its caudal slice set at the level of basal ganglia. In this way, the scanning area was able to assess perfusion in anterior, middle, and posterior cerebral artery vascular territories. Simultaneously to intravenous contrast injection (50 ml of nonionic iodinated contrast material at a rate of 5ml/s), 50 cycles 1/s on a package of 24mm were acquired, producing 200 raw images.

GE Advantage Workstation using CTP software version 3.0 or 4D (GE Medical Systems) was used to postprocess the parametric maps. The arterial input function (AIF) was set on the anterior cerebral artery, while the venous input function was set on superior sagittal sinus on the same scanning plane.

**Figure 1:**
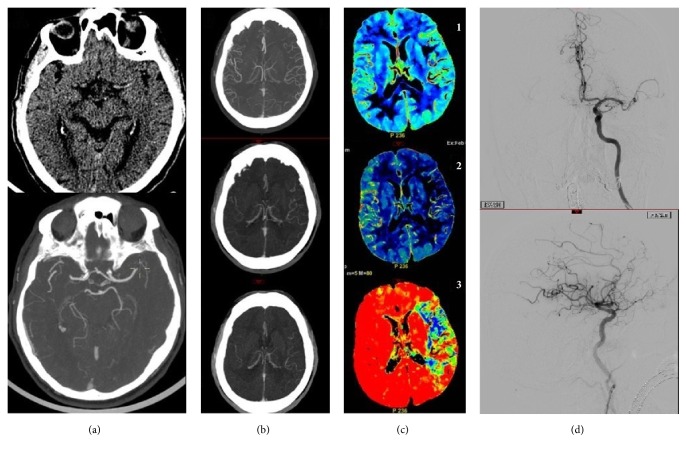
82-year-old patient, smoker, presenting at 2 hours from symptoms onset with an NIHSS score of 20. (a) CT shows left hyperdense MCA, CTA absence of flow in the same MCA region. (b) Multiphase CTA shows good collaterals in the arterial phase and two venous phases of acquisition. (c) CTP shows mismatch between the large area on MTT (3) and the small infarct core on CBV (1). (d) Angiography shows an occluded MCA with perfect recanalization (TICI 3).

Volumes of hypoperfused territories, ischemic core, and penumbra area were assessed by analyzing Cerebral Blood Volume (CBV), Mean Transit Time (MTT), and Cerebral Blood Flow (CBF) maps (Figure 1).

### 2.4. Endovascular Procedure

Endovascular therapy, using ADAP-technique, was carried out as described thoroughly in other works [11, 12].

After femoral puncture a large guide catheter [Penumbra Neuron 088 Max (Penumbra Inc., Alameda, CA, USA)] was navigated to the carotid bulb. The aspiration catheter, chosen according to patient anatomy and site of vessel occlusion (Penumbra 5MAX ACE or 4MAX or 3MAX or ACE64 or ACE68; Penumbra Inc., Alameda, CA, USA) was then advanced proximally to the thrombus, usually with triaxial technique using a microcatheter (Velocity, Penumbra Inc., Alameda, CA, USA) and a microguidewire (Synchro, Striker Neurovascular, Kalamazoo, MI, USA) which were subsequently removed to begin the aspiration using the Penumbra aspiration pump. In three patients, it was necessary to deploy a carotid stent (XAct, Abbott, Chicago, Illinois, USA) followed by angioplasty inside the stent (Ultrasoft, Boston Scientific, Marlborough, Massachusetts, USA) to treat stenosis of Internal Carotid Artery (ICA). Patients that required stenting were administered single antiplatelet therapy the same day (100 mg Acetyl Salicylic Acid) while double antiplatelet therapy was administered only after 24h in NCCT excluded hemorrhages.

### 2.5. Clinical Evaluation

Age, Sex, side of lesion, and stroke risk factors were registered. Age was then stratified into ≥80 and <80 as a cutoff seen, due to reported differences in literature regarding clinical outcomes after thrombectomy [13].

Time from onset of symptoms to hospital, to CT, to needle (IV tPA), to groin (femoral artery puncture), and to end of procedure were noted.

A premorbid modified Rankin Scale (mRS) was calculated and used as a measure of disability and functional limitation before the event [14]; mRS was then collected at discharge and at 90 days through telephone questionnaires [15].

NIHSS was collected by an expert neurologist to evaluate stroke severity at admission and discharge. It was then stratified as severe or moderate with a 15-point cutoff both at admission [16] and at discharge and used to measure the consistency of imaging with clinical data.

### 2.6. Outcome Evaluation

Various imaging scores present in literature and proven to be predictive of good outcome were collected for each patient and then stratified according to known [17] cutoffs to test their outcome predictivity in our sample (Table 2).

Good outcome was considered as a mRS≤2 at discharge and at 90 days [18], or an improvement of NIHSS >8 from admission to discharge.

Thrombolysis in Cerebral Infarction (TICI) classification was calculated by the interventional radiologist after each treatment to assess the degree of vessel occlusion. A TICI score of 2b or 3 identified a successful revascularization [19].

A discharge NIHSS >14 was also included as an outcome to be used as an acute marker for higher death and dependency risk.

### 2.7. Statistical Analysis

All analyses were performed using SPSS (Version 20.0. SPSS Inc., Chicago, IL, USA). Descriptive results and quantitative baseline patient characteristics were reported as mean ± SD or median (IQR), ratios, as percentage.

Nonparametric statistical tests were applied to the sample being less than 30. Wilcoxon rank-sum (Mann–Whitney) test was applied to compare continuous data with outcomes expressed as dichotomic variables; Fisher Exact Test was used to obtain* p *values out of contingency tables instead.

## 3. Results

30 patients met the inclusion criteria and were treated for ELVO with ADAPT. Mean age of patients was 71.5 ± 15.34 (min-max 18-94) with 8% of subjects being over the age of 80; 40% were male and 63% had a left sided stroke. Mean time from symptom onset to reaching Novara Hospital (Hub) or a neighboring (Spoke) hospital was 85±55min (range: 30-300); time to CT scan was 120±63min (10-320), to IV tPA was 192±62min (100-358), and to groin needle puncture (procedure initiation) was 250±55min min (range 100-358). Mean procedural time was 43 minutes (range 27-112). Twenty-two (73%) patients received IV tPA; TICI 2b or 3 revascularization was obtained in 17 patients (57%). 24hrs CT showed Ph1 hemorrhage in 2 patients (6%).

Mean admission NIHSS score was 17±5.05 (range 6-24) while mean discharge NIHSS was 9±5.96 (0-19) with a mean improvement of 10±7.7 (-2-24).

Mean premorbid mRS was 1.07±1.14 (0-3), while discharge mRS was 4.07±1.91 (0-6) and 3.71±2. 175(0-6) at 90 days. Mortality was 23% (7) at discharge and 30% (9) at 90 days. Good outcome expressed a mRS ≤2 or an improvement in NIHSS >8 was obtained in 11 patients (37%) (Table 3).

Tests were considered statistically relevant given a significance level (*α*) of 0.05.

Patients with severe stroke on admission (NIHSS>15) were not significantly more likely to have a lower ASPECT score on NCCT median (IQR) (7 (7-9) versus 9 (7-9); p=0.27), a lower ASPECT on CTP (7 (6-9) versus 8.5 (8-10); p=0.10), lower CBS (p=0.40), or worse collaterals on single phase CTA (p=0.24) than those having a moderate to mild stroke. Neither did the presence of leukoaraiosis (p=0.11) and clot density ratio (p=0.17).

Higher core volume on CTP (p=0.015), bigger penumbra volume (p=0.03), and a clot length more than 10mm (p=0.017) were statistically associated with a higher NIHSS at admission.

Other factors were significantly correlated to worse NIHSS score: people presenting with a higher number of comorbidities and worse functionality were represented by a higher premorbid mRS (2+) (p=0.001) and in a longer time to hospital (p=0.036) had worse scores than others (Table 4).

Neither clinical data nor imaging scores could predict revascularization (TICI 2b-3) with statistical significance. Not even collaterals as PAF on single phase CTA (p=0.12) nor time to IV tPA (p=0.10) was associated with a higher revascularization rate.

Patients were more likely to have a severe NIHSS at discharge (>15) when presenting with low ASPECT at CTP (p=0.03), low CBS (p=0.04), bad collaterals (PAF) (p=0.025), and long time from symptoms onset to IV tPA (p=0.04) rather than a moderate-mild stroke (Table 4).

Good outcome was significantly more likely to be associated with a better TICI score (p=0.007) and ASPECT score on NCCT (p=0.05). In our sample, we could not prove that ASPECT on CTP (p=0.34), core volume (p=0.08), penumbra volume (p=0.06), collaterals as PAF on single phase CTA (p=0.13), or leukoaraiosis (p=0.14) would determine a statistical difference in outcome groups (Table 5).

The 90-day good outcome (mRS ≤2) was statistically associated with lower premorbid mRS (p=0.01), higher ASPECT score on NCCT (p=0.048), and clot length <10mm (p=0.05).

No other variables met the intended p value; clot density ratio (p=0.06), time to needle (p=0.17), TICI (p=0.09), and sex (p=0.09) were not determinant to a better long-term outcome (Table 5).

## 4. Discussion

Recent large multicenter trials have demonstrated the value and importance of endovascular therapy for the treatment of anterior circulation ischemic strokes. New materials used in these treatments are produced at a high rate and keeping up with the technological development is becoming always more challenging. MRI is considered to be the most specific and sensitive tool in diagnosis and patient selection in ischemic stroke, but it is not available in every facility, it is sometimes hard to be performed in emergency settings and it is time consuming.

Therefore, a correct protocol of CT imaging could be a good alternative for these purposes. 16-slice multidetector-row CT scanners are widespread instruments in hospitals. CTA with brain scans should be a service that not only every Hub but also each Spoke can give. Also understanding the presence of penumbra on CTP thanks to colored maps is something that every trained radiologist can do. Finding scores able to select patients eligible for endovascular therapy and being able to send them as soon as possible to an interventional radiology department are of utmost importance.

Until recently, intravenous thrombolysis with rtPA was the only available drug therapy of stroke patients [20]. This treatment can be administered within the first 4.5 hours after symptom onset and only if a hemorrhagic etiology has been excluded by NNCT or MRI [6].

Intravenous thrombolysis has been demonstrated to have a lower efficacy in patients with ELVO, showing a recanalization rate ranging from 8% (in patient with ICA proximal occlusion) up to 26% (in M1 occlusions) [21, 22]. Around one-third of cases of acute ischemic stroke have been demonstrated to be caused by occlusion in proximal anterior circulation (involving ICA, M1, or M2 segments) which are affected by a worse prognosis if treated only with tPA [23].

This study shows the feasibility of this patient selection and treatment methods in a center that has just begun to perform endovascular therapy.

We had 8% of cases over 80 years, in a sample with a mean age of 71 years old; 73 % of cases had been carried out with previous IV fibrinolytic therapy. Good outcome was achieved in 37% of patients. Mortality was 23% at discharge and 30% at 3 months, being increased by myocardial infarction (2) and pneumonia (1). Among whole case series, we had a rate of 6% of hemorrhagic transformation of the ischemic area, which involved less than 30% of the infarct zone (Ph1).

Mean time required to get to CT/vein/groin in our sample shows the need for improvement of stroke diagnosis and treatment paths; in our case series time needed to perform CT scan was too long, being in average around 2 hours, showing need of improvement in the management of our next patients.

A strong statistical correlation was found between imaging scores on CTP (as presence of large core volumes, bigger penumbra volumes, and clot length (>10 mm) on NCCT) with stroke severity at admission. In our case series, clinical scores such as premorbidity conditions and longer time spent to arrive to the hospital were a determinant factor for initial severity.

Multimodal imaging, especially NCCT and CTP, appeared therefore to be a good and precise tool to assess clinical severity of the stroke. This is to be considered an important tool in patient selection especially in those patients whose clinical conditions do not allow a correct neurological evaluation (e.g., intubated patients).

Surprisingly, time to CT, that we consider long in our case series, did not show a correlation with a worse NIHSS score at admission (Table 4). This could be explained by the fact that patients with higher NIHSS show more prominent symptoms that could be evaluated promptly leading to a fasted hospital admittance.

NCCT images could already provide useful information on patient outcome, especially with ASPECT score assessment; as reported in literature [24] an ASPECT score <7 is an important predictor of unfavorable outcome with a linear inverse relationship between ASPECTS at baseline and functional outcome [25, 26]; in our study, we reported a similar conclusion with ASPECTS on NCCT at baseline, as it was statistically related to patient's outcome.

In our case series clot length assessed on NCCT appeared to be statistically associated with a good outcome at 90 days; similar findings were reported by Riedel et al. [27], which stated that clot length estimated at NCCT could predict patient who could benefit less from IV thrombolysis as thrombus length greater than 8mm is usually associated with poor recanalization and outcome; this finding might suggest that shorter thrombi are easier to remove by aspiration alone, leading to a better clinical outcome.

In our study, we did not find any score, obtained at CTA imaging, statistically related to good outcome at discharge or at 90 days; this result could be explained by the small sample of patients selected in our study. Nevertheless, multiple studies have demonstrated the correlation of CTA scores, such as Pial Arterial Filling Score [8] and Clot Burden Score [28], with recanalization rate and outcome. In our case series, we reported a slightly more favorable outcome in patients with good PAF and high CBS; even if this correlation did not reach a statistical significance (PAF p=0.1 and CBS p=0.13), it is a good hint for improvement and widening of this study to better detect patients that could benefit from the treatment.

Campbell BCV et al. [29] reported that a core infarct volume of 70ml, calculated in CTP images, is usually the upper limit above which poor outcome is expected. Even though in our study no statistical correlation was found, there was a trend of more favorable outcome in patients with a smaller infarct core volume and penumbra volume, in agreement with Campbell's study.

Our data confirmed the relevance of NCCT imaging (both ASPECT and clot length) in predicting clinical severity and outcome. Even though scores found at CTA and CTP did not reach statistical significance, we witnessed a trend of better outcome in patients with good scores confirming the importance of having a good and standardized imaging protocol.

Both DEFUSE-3 and DAWN trials [4] confirmed the diagnostic power of perfusion CT, confirming that in selected patients, recanalization of the occluded vessel leads to clinical improvement even well past beyond the previously stated 6-hour time window.

In DEFUSE-3 [4] all patients were evaluated with the use of RAPID software (iSchemaView), an automated image postprocessing tool, which can easily estimate size of penumbra and core on TMax maps. DAWN trial design was different and allowed single centers and operators to use different tools to evaluate core and penumbra; both MRI and CTP were used, but for size analysis postprocessing was always performed with RAPID software. In our institution, RAPID software is not available; therefore CTP images were postprocessed with GE perfusion software and analyzed by a single expert neuroradiologist to avoid inter-operator variability. Taking into account these limitations in our study, we were still be able to find some trend of improvement in patient with good CTP findings.

Clinical scores such as premorbidity (pre-mRS) and time to needle (IV tPA) were significantly correlated to NIHSS at discharge; this finding underlines how IV thrombolysis is still a useful therapy for stroke patients, especially if performed early from the onset, even in patient that are subsequently treated with mechanical thrombectomy.

Recent randomized trials [24–27, 30–38] have demonstrated the efficacy of thrombectomy in patient with ischemic stroke due to emergent large vessel occlusion (ELVO) within a time window that was set at 6 hours after symptoms onset. In these trials imaging findings were used to exclude patients from treatment rather than recruit them; on the contrary in our study we analyzed imaging results trying to extend patients inclusion even beyond time window and even if clinical conditions of the patients were not optimal, widening the number of patients eligible for treatment. Thrombectomy devices continued to evolve in recent years, and endovascular procedure now can rely on different tools and approaches, such as stent retriever (metallic stent opened inside the occluded vessel and retrieved to extract the occluding thrombus) [22, 33–35] or thromboaspiration catheters which, after being attached to an aspiration pump, can suck out the thrombus to restore the vessel patency [11, 36, 37].These techniques can also be applied together, having a thromboaspiration catheter proximally to the deployed stent; this solution creates a slower but safer procedure because the stent retriever is pulled into the aspiration catheter in the occluded vessel, reducing the risk of distal emboli to new territories and minimizing traction on the cerebral vessels.

Improvements in catheter technology have resulted in decreasing procedure times, using only thromboaspiration catheters with ADAPT, of 30 to 40 minutes on average [11], nearly half of time required with stent retrievers [12, 38].

ADAPT is a fast and safe technique to obtain revascularization, which is also simple, needing only a flexible large bore catheter to aspirate thrombi after navigation of intracranial large vessels.

Results reported in our case series were close to those found in the literature considering outcome and mortality [11, 25, 30–34, 39]. Reasons for discrepancies from other literature and prior studies could be various. Probably, the small sample in which few cases affect those values close to statistical significance. One main reason could be the selection criteria used in those trials, which excluded patients with a premorbid mRS>1. Using the logic behind the theory that mismatch between penumbra and ischemic core on CTP represents salvageable brain tissue, we included patients that would have been excluded in those trials. Two patients out of nine (22%) of those that would have been excluded by strict inclusion criteria from randomized studies had a good outcome. We also found it hard to compare outcomes using mRS, since a patient with premorbid mRS of 3 could not achieve an mRS≤2 by definition, making it necessary to use some other kind of score to compare clinical functionality before and after the stroke, affecting the overall clinical success of the population in study. Another reason could be that patients analyzed in our study were all treated with only ADAP-technique, without deploying stent retriever in case of unsuccessful recanalization after thromboaspiration.

As reported in our study, times of patient transfer, from Spoke to hospital, from CT to needle, and to groin are slightly longer than the ones reported in literature [30–36]. This could be explained by several causes. First of all, the territory of our Hub hospital is quite large, serving more than one million inhabitants, with several Spoke hospitals scattered around the area, which require long times of centralization. Secondly, due to intrahospital protocol, IV fibrinolysis must be administered in stroke unit setting, to allow weighing of the patients and the management of blood pressure; therefore, two transfers are required for patients that are treated with combined therapy. Surely patient transfer optimization is a key-point in stroke patient management, as stated by Khatri et al. [40]; each 30 minutes of delay to recanalization leads to a reduction of 10% of good outcome. This study is a report of the first 2 years of our experience in stroke thrombectomy, and it provides useful hints for improvement of our internal protocols.

Multimodal CT/CTA/CTP is a fundamental, safe, and relatively inexpensive method to evaluate correctly the severity of ischemic stroke. It gives the necessary information to set up the correct therapy to achieve a good outcome for every patient. Scores found in the literature, such as ASPECT on NCCT and CTP, measuring clot density, checking for leukoaraiosis, collaterals, mismatch area, and ischemic core and penumbra volumes on CTP, are tools that could help the radiologist decide whether a patient would truly benefit from an endovascular procedure.

Endovascular therapies proved to be safe and, in the future, they could be performed also in smaller centers, in order to cover a larger geographical area, granting faster treatment times. ADAPT used in our study appeared to be a safe and effective way of treatment, particularly since procedural times appear to be substantially reduced if compared to other technique.

Patients presenting with a premorbid state worse than what is now accepted for treatment could still benefit from endovascular therapy when selected with appropriate imaging criteria.

Defining a “good outcome” as a poststroke mRS≤2 excluded in previous large trials a priori endovascular therapy in patients with a high premorbid mRS. Our study demonstrates that including severely ill patients with favorable imaging criteria increases the number of those who would benefit from mechanical thrombectomy, reducing death, worse clinical functionality, and need for poststroke care.

## Figures and Tables

**Table 1:** Clinical and imaging inclusion and exclusion criteria of patient.

**Inclusion Criteria**	**Exclusion Criteria**
*Clinical:* (i) Age >18 years (ii) Time from onset <6 hours (iii) NIHSS ≥5 at presentation	*Clinical:* (i) Concurrent myocardial infarction or severe infection (endocarditis or sepsis) (ii) Uncontrollable hypertension defined as systolic blood pressure >185mmHg or diastolic pressure > 110mmHg (iii) Life expectancy of less than 90 days before stroke onset (iv) Pregnant or lactating women (v) Known severe allergy to radiographic contrast medium (vi) Improvement of NIHSS score >4 in less than 1 hour
*Imaging:* (i) Arterial occlusion of distal Internal Carotid Artery (ICA) or Middle Cerebral Artery (M1/M2) (ii) ASPECT-score on NCCT ≥6 (iii) Evidence of favorable criteria on CTA or CTP or Collaterals (iv) Wide Mismatch between Mean Transit Time (MTT) and Cerebral Blood Volume (CBV)	*Imaging:* (i) CT evidence of significant mass effect with midline shift (ii) CT evidence of intracranial hemorrhage (ICH), Subarachnoid Hemorrhage (SAH), Aneurism or Cerebral arteriovenous malformations (CAVMs).

**Table 2:** Scores and parameters evaluated on Noncontrast Computed Tomography (NCCT), Computed Tomography Angiography (CTA), and Computed Tomography Perfusion (CTP).

NCCT	CTA	CTP
(i) ASPECTS, stratified *≤7 and >7 * (ii) Leukoaraiosis, stratified as “present” or “absent” (iii) Clot Length, divided >10mm or <10mm (iv) Clot Density (Hounsfield Units) corrected by contralateral density: Clot density ratio (v) Site of Occlusion	(i) Clot Burden Score (CBS), stratified *≥6 and <6 * (ii) Collaterals as Pial Arterial Filling Score (PAF) within the affected territory Using Single and Multiphase CT Angiography. Stratified in >4 and ≤4	(i) Ischemic Core Volume, Penumbra Volume using the ABC/2 formula (ii) Mismatch between Mean Transit Time (MTT) and Cerebral Blood Volume (CBV) (iii) CBV ASPECT, then stratified in >8 and ≤8

**Table 3:** Clinical and demographic variables, imaging characteristics, and outcomes.

Total # of patients	30
Sex (M/F)	18/12
Left Hemisphere	19/30 (63%)
Age	71.5 ± 15.34
NIHSS at admission (range)	17 (14-20)
IV tPA treatment	22/30 (73%)

Location of the occlusion:	

Tandem (ICA and M1 segment)	14/30 (47%)
ICA	4/30 (13%)
MCA, M1 segment	10/30 (33%)
MCA, M2 segment	2/30 (7%)

Timings:	

Time onset to admission (hours)	1.4 (0.96-1.6)
Time to CT	2 (1.4-2.3)
Time to IV-tPA	3.2 (2.4-3.8)
Time to groin	4.2 (3.4-4.8)

Clinical and Radiological Outcomes:	

Good Outcome	11/30 (37%)
Mortality	7/30 (23%)
TICI (2b-3)	17/30 (57%)
PH2 incidence	2/30 (7%)

**Table 4:** Correlation with stroke severity at admission(*a*) and at discharge (*b*). Severe stroke as NIHSS >15; moderate ≤15; †Fisher exact test; °Wilcoxon (Mann–Whitney U) test.

*A*		**N tot**	**Severe stroke**	**N**	**Moderate-mild stroke**	**N**	**P value**
**ASPECT NCCT**°		28	7 (7-9)	[17]	9 (7-9)	[11]	0.27
**CBV ASPECT CTP**°		23	7 (6-9)	[13]	8.5 (8-10)	[10]	0.10
**CBS**°		29	5 (3-6)	[18]	6 (3-8)	[11]	0.40
**PAF Score Single Phase CTA**°		28	4 (3-4.5)	[17]	4 (4-5)	[11]	0.24
**Leukoaraiosis†**	Present	29	7 (39%)		1 (9%)		0.11
	Absent		11 (61%)		10 (91%)		
**Clot Density Ratio**°		28	1.45 (1.3-1.7)	[17]	1.36 (1.26-1.44)	[11]	0.17
**CTP Core Volume**°** (mm**^**3**^**)**		23	20 (8-34)	[13]	2 (1.5-8)	[10]	0.015^*∗*^
**CTP Penumbra Volume**°** (mm**^**3**^**)**		23	51 (42-63)	[13]	68 (52-89)	[10]	0.03^*∗*^
**Clot Length†**	≥10mm	29	15 (83%)		4 (36%)		0.017^*∗*^
	≤10mm		3 (17%)		7 (64%)		
**Premorbid mRS**		30	1 (1-2)	[19]	0 (0-0)	[11]	0.001^*∗*^
**Time to hospital**°** (min)**		29	67 (56-81)	[18]	90 (60-130)	[11]	0.036^*∗*^

*b*		**N tot**	**Severe stroke**	**N**	**Moderate-mild stroke**	**N**	**P value**

**ASPECT CTP**°		18	8.5 (7.5-9.5)	[16]	5.5 (5-6)	[2]	0.03^*∗*^
**ASPECT NCCT**°		22	9 (7.5-9)	[20]	7 (7-7)	[2]	0.12
**ASPECT NCCT stratified**°	>8	21	15 (75%)		0 (0%)		0.09
	≤8		5 (25%)		1 (100%)		
**CBS**°		22	5 (3.5-7)	[20]	2.5 (2-3)	[2]	0.04^*∗*^
**PAF Score Single Phase CTA**°		21	4 (4-5)	[19]	3 (3-3)	[2]	0.025^*∗*^
**Clot Density Ratio**°		21	1.36 (1.26-1.44)	[19]	1.45 (1.44-1.46)	[2]	0.23
**CTP Core Volume**°** (mm**^**3**^**)**		18	4.25 (1.75-23)	[16]	50 (40-60)	[2]	0.04^*∗*^
**CTP Penumbra Volume**°** (mm**^**3**^**)**		18	78.5 (65.5-88)	[16]	103 (91-115)	[2]	0.08
**Mismatch†**	≥50%	18	15 (94%)		0 (0%)		0.02^*∗*^
	<50%		1 (6%)		2 (100%)		
**Leukoaraiosis†**	Present	22	2 (10%)		1 (50%)		0.26
	Absent		18 (90%)		1 (50%)		
**Premorbid mRS**		23	0 (0-1)	[20]	2 (1-2)	[3]	0.09
**Time to needle**°** (min)**		18	180 (150-244)	[15]	140 (100-156)	[3]	0.04^*∗*^

**Table 5:** Correlation with outcome at discharge (*a) *and at 3 months (*b*). Good outcome intended as improvement of NIHSS >8 from admission to discharge or a mRS≤2 at discharge. †Fisher exact test. °Wilcoxon (Mann–Whitney U) test. Data expressed as median (IQR).

*a*		**N tot**	**Good outcome**	**N**	**Bad outcome**	**N**	**P value**
**ASPECT CTP stratified†**	>8	18	3 (30%)		5 (62.5%)		0.34
	≤8		7 (70%)		3 (37.5%)		
**ASPECT NCCT**°		28	9 (8-10)	[7]	7 (7-9)	[21]	0.049^*∗*^
**CBS**°		29	6 (5-7)	[7]	5 (3-7)	[22]	0.15
**PAF Score Single Phase CTA**°		28	4 (4-5)	[7]	4 (3-4)	[21]	0.13
**CTP Core Volume**°** (mm**^**3**^**)**		23	3.25 (1.5-5)	[6]	15 (4.5-15)	[17]	0.08
**CTP Penumbra Volume**°** (mm**^**3**^**)**		18	55 (42-66)	[10]	71.5 (57.75-89)	[8]	0.06
**Leukoaraiosis†**	Present	29	0 (0%)		8 (36%)		0.14
	Absent		7 (100%)		14 (64%)		
**TICI Score†**	2b-3	30	10 (91%)		7 (37%)		0.007^*∗*^
	0.2a		1 (9%)		12 (63%)		

*b*		**N tot**	**Good outcome**	**N**	**Bad outcome**	**N**	**P value**

**NIHSS at entrance†**	>15	28	4 (40%)		15 (75%)		0.11
	≤15		6 (60%)		5 (25%)		
**ASPECT NCCT**°		28	8 (8-9)	[20]	7 (7-9)	[18]	0.048^*∗*^
**Premorbid mRS**°		30	0 (0-1)	[10]	2 (0-2)	[20]	0.01^*∗*^
**Clot Density Ratio**°		27	1.37 (1.25-1.54)	[7]	1.43 (1.29-1.62)	[20]	0.06
**Time to needle**°** (min)**		22	163 (140-232)	[6]	187 (175-200)	[16]	0.17
**Sex†**	M	28	7 (85.5%)		10 (50%)		0.099
	F		1 (12.5%)		10 (50%)		
**Clot Length†**	>10	28	4 (40%)		15 (79%)		0.05^*∗*^
	≤10		6 (60%)		4 (21%)		
**TICI Score†**	2b-3	28	7 (87.5%)		10 (50%)		0.099
	0-2a		1 (12.5%)		10 (50%)		

## Data Availability

The stroke patients and CT data used to support the findings of this study are available from the corresponding author upon request.
